# *TIGIT* Monoallelic Nonsense Variant in Patient with Severe COVID-19 Infection, Thailand

**DOI:** 10.3201/eid2811.220914

**Published:** 2022-11

**Authors:** Pimpayao Sodsai, Chupong Ittiwut, Vichaya Ruenjaiman, Rungnapa Ittiwut, Watsamon Jantarabenjakul, Kanya Suphapeetiporn, Vorasuk Shotelersuk, Nattiya Hirankarn

**Affiliations:** Chulalongkorn University, Bangkok, Thailand (P. Sodsai, C. Ittiwut, V. Ruenjaiman, R. Ittiwut, W. Jantarabenjakul, K. Suphapeetiporn, V. Shotelersuk, N. Hirankarn);; King Chulalongkorn Memorial Hospital, The Thai Red Cross Society, Bangkok (W. Jantarabenjakul, R. Ittiwut)

**Keywords:** COVID-19, respiratory infections, severe acute respiratory syndrome coronavirus 2, SARS-CoV-2, SARS, coronavirus disease, zoonoses, viruses, coronavirus, TIGIT, T cells, Thailand

## Abstract

A heterozygous nonsense variant in the *TIGIT* gene was identified in a patient in Thailand who had severe COVID-19, resulting in lower *TIGIT* expression in T cells. The patient’s T cells produced higher levels of cytokines upon stimulation. This mutation causes less-controlled immune responses, which might contribute to COVID-19 severity.

To investigate SARS-CoV-2 genomic variants, we recruited 46 COVID-19 patients from King Chulalongkorn Memorial Hospital in Bangkok, Thailand, in January 2020. Recruited patients were 16–79 years of age and had moderate to severe COVID-19 symptoms according to World Health Organization interim guidelines (https://apps.who.int/iris/bitstream/handle/10665/331446/WHO-2019-nCoV-clinical-2020.4-eng.pdf). We performed whole-exome sequencing on peripheral blood samples as described (*1*). The institutional review board of the Faculty of Medicine, Chulalongkorn University, Bangkok, approved this study (COA no. 738/2020). 

We filtered variants by using the following criteria. Variants had to pass the quality standards, have read depth >10, and be from the coding regions or canonical splice sites of 1,810 immune-related genes, including immune checkpoint genes (*2*). Variants also had to have <1% allele frequency in the Genome Aggregation Database (gnomAD, https://gnomad.broadinstitute.org), Exome Variant Server (University of Washington, https://evs.gs.washington.edu/EVS), 1000 Genomes Project Consortium (https://www.genome.gov), dbSNPs (https://www.ncbi.nlm.nih.gov/projects/SNP), and Thai Reference Exome (T-Rex) database (*3*). We called candidate variants novel pathogenic variants when they were not previously identified in patients in the literature.

In our patient cohort, exome sequencing identified no variants in type I interferon genes, which previously have been commonly observed in patients with severe COVID-19 (*4*). Of note, we identified a heterozygous nonsense variant (rs1386709957) in the T-cell immunoglobulin and ITIM domain (*TIGIT*) gene in 1 patient (Appendix Figure 1). We did not identify this nonsense variant among 3,742 persons in the T-Rex database but did observe it in 1 of 31,390 alleles in the gnomAD database, in an allele from a female patient from East Asia. This variant truncates the 245-amino acid residue proteins at residue 56 and is classified as a pathogenic variant American College of Medical Genetics guidelines (https://www.acmg.net). 

We investigated *TIGIT* gene expression in T cells of the patient from our study (Co45), a 43-year-old man, and compared it with 2 other sex- and age-matched patients who had severe COVID-19 (Co6 and Co84) (Appendix). We collected peripheral blood mononuclear cells (PBMCs) from each of the patients 1 month after they recovered. We used RNA extracted from PBMCs for real-time reverse transcription PCR and found patient Co45 had the lowest *TIGIT* mRNA level (Figure, panel A). Because *TIGIT* is mainly expressed in T cells, we used flow cytometry to measure the mean fluorescence intensity of TIGIT expressed in the cytoplasmic domain (CD) T cells. Patient Co45 had lower TIGIT gene expression in all CD3+, CD4+, and CD8+ T cells than the other 2 patients, most remarkably in the CD8+ T cells (Figure, panels B–D). The percentages of CD3+, CD4+, and CD8+ T cells in patient Co45 were comparable those in the other 2 patients (Appendix Figure 2, panel A), demonstrating that the truncated *TIGIT* variant reduced *TIGIT* expression in CD3+, CD4+, and CD8+ T cells.

**Figure:**
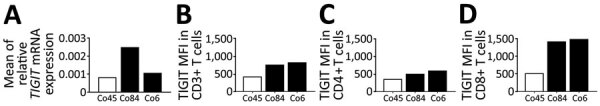
Results of rRT-PCR assay and flow cytometry of *TIGIT* nonsense variant in a patient with severe COVID-19 infection, Thailand. Co45 is the patient with *TIGIT* nonsense variant; Co84 and Co6 are age- and sex-matched patients who also had severe COVID-19 infection. A) Mean relative mRNA levels of *TIGIT* expression from rRT-PCR assay. B) TIGIT expression MFI on CD3+ T cells. C) TIGIT expression MFI on CD4+ T cells. D) TIGIT expression MFI on CD8+ T cells. CD, cytoplasmic domain; MFI, mean fluorescence intensity; rRT-PCR, real-time reverse transcription PCR; *TIGIT*, T cell immunoglobulin and ITIM domain gene.

*TIGIT* is known to exert immune suppressive functions, such as inhibiting T cell activation, proliferation, and functions that inhibit inflammation and anti-tumor responses. Thus, we investigated the effect of this monoallelic *TIGIT* variant on T cell functions by examining activation markers and cytokine-secreting T cells after stimulation with anti-CD3/CD28 coupled beads for 24 hours. We then assessed activation by using flow cytometry. We found no differences in frequencies of CD69-expressing CD3+, CD4+, and CD8+ T cells among the 3 patients (Appendix Figure 2, panel B); however, patient Co45 had higher interferon gamma (IFNγ), tumor necrosis factor alpha (TNF-α), and interleukin (IL) 2–producing CD3+, CD4+, and CD8+ T cells than the other 2 patients (Appendix Figure 3). 

We believe this patient’s heterozygous nonsense *TIGIT* variant contributed to the increased inflammatory cytokine functions we observed. His serum cytokine levels at acute illness onset did not differ from the other 2 COVID-19 patients (Appendix Figure 4), but some of his cytokine levels, including IL-10, IL-12p70, IL-4, and IL-7, remained high 1 month after recovery. Upregulation of co-inhibitory receptors, including cytotoxic T-lymphocyte–associated protein 4, programmed cell death protein 1, lymphocyte-activation gene 3, and T-cell immunoglobulin mucin-3, including *TIGIT*, has been reported in COVID-19 patients in other studies (*5*). These co-inhibitory receptors upregulated after T-cell activation to regulate immune responses and limit immunopathology (*6*,*7*). *TIGIT* can modulate expression of proinflammatory cytokines in acute lymphocytic choriomeningitis virus infection, in which the *TIGIT* blockage increased TNF-α expression by CD8+ T cells (*8*). *TIGIT* -deficient mice displayed increased IFNγ and IL-17+CD4+ T-cell frequencies (*9*). Similarly, *TIGIT* knockdown can increase IFNγ expression in human T cells (*10*). We hypothesize that the nonsense *TIGIT* variant led to low *TIGIT* expression and hyperactive T responses in patient Co45 and might have contributed to his severe inflammation and symptoms. Unfortunately, the patient refused follow-up, so we could not perform further investigations to confirm our hypothesis.

In conclusion, we identified a patient with severe COVID-19 and a *TIGIT* monoallelic nonsense variant. He had lower *TIGIT* expression in CD3+, CD4+, and CD8+ T cells and produced higher cytokine expression, including IFNγ, TNF-α, and IL-2 upon stimulation. Our findings suggest *TIGIT* could be involved in COVID-19 severity.

Appendix Additional information on *TIGIT* nonsense variant in a patient with severe COVID-19 infection, Thailand.
