# Supplementary material for: TIGIT Monoallelic Nonsense Variant in Patient with Severe COVID-19 Infection, Thailand
Source: Emerg Infect Dis. 2022 Nov;28(11):2350–2. doi: 10.3201/eid2811.220914 (PMC9622227; doi:10.3201/eid2811.220914)
Supplement: Appendix — Additional information on TIGIT nonsense variant in a patient with severe COVID-19 infection, Thailand. [file 22-0914-Techapp-s1.pdf]

Article DOI: https://doi.org/10.3201/eid2811.220914

TIGIT Monoallelic Nonsense Variant in Patient with Severe COVID-19 Infection, Thailand

Appendix

Materials and Methods

Subject Recruitment and Sample Collection

In January 2020, we recruited 46 patients with moderate to severe COVID-19 from the King Chulalongkorn Memorial Hospital who were confirmed by standard real-time reverse transcription PCR for SARS-CoV-2 from nasopharyngeal swabs. COVID-19 severity was classified as follows: mild for mild symptoms or only upper respiratory tract infection; moderate for pneumonia without oxygen desaturation; or severe for pneumonia treated with oxygen support. This study was reviewed and approved by the institutional review board of the Faculty of Medicine, Chulalongkorn University (COA no. 738/2020). In addition, written informed consent was obtained from all patients.

We enrolled 1 patient (Co45) with NM_173799.4:c.166C>T (hg19, chr3:114014496 C>T, p.Gln56Ter, rs1386709957) variant in the T cell immunoglobulin and ITIM domain (*TIGIT*) gene and 2 patients (Co6 and Co84) without the variant, all of whom had severe COVID-19 symptoms. We collected serum samples at the acute illness onset or diagnosis, on various hospitalization days (day 4, 5, or 6), and again 1 month after recovery or hospital discharge. In addition, we also collected peripheral blood mononuclear cells (PBMCs) from these 3 patients (Co45, Co6, Co84) at 1-month post-recovery to examine the phenotyping and function of T cells. For healthy controls, we collected blood from 40 healthy donors from the Thai Red Cross Society (IRB approval no. 426/63). All healthy donors were negative for the IgG test kit for SARS-CoV-2.

Clinical Data of Patient with *TIGIT* Variant

A 43-year-old male patient with *TIGIT* nonsense variant (patient Co45) had a history of pulmonary tuberculosis 10 years prior and presented with cough, nasal congestion, and myalgia. At admission, his physical examination revealed body mass index of 24 kg/m^2, body temperature of 36.5°C, respiratory rate 18/min, SpO_2 99%, with normal breath sounds. Chest x-ray showed minimal and faint reticular opacity in both upper lungs, and no cavity was noted. Complete blood count revealed hematocrit of 49.6%, white blood count 5,710 cells/mm^3 (PMNs 59%, lymphocytes 30.8%), and normal platelet count (222,000/mm^3). After 5 days of admission, a new low-grade fever developed with dyspnea (SpO2 95% on room air), and new patchy opacity appeared in the right lower lung field. He was treated with favipiravir 1,600 mg twice per day for the first day and 600 mg twice per day for the next 9 days, oral azithromycin 500 mg daily for the first day, then 250 mg daily for the next 4 days, and hydroxychloroquine 200 mg 3 times per day for 10 days according to a regimen for pneumonia in March 2020. Three days later, his symptoms improved and was afebrile and had normal oxygen saturation during the 6-minute walk test.

Exome Sequencing

Peripheral blood was obtained for genomic DNA extraction, then whole-exome sequencing (WES) of all 46 patients was performed, as previously described (*1*). In brief, the sequencing libraries were enriched by using a SureSelect Human All Exon V7 Kit (Agilent Technologies, https://www.agilent.com) and were sequenced using HiSeq 4000 (Illumina, https:www.illumina.com). The variants were filtered with the following criteria: 1) passed the quality filters; 2) had read depth >10; 3) located in the coding regions and canonical splice sites of 1,810 immune-related genes, including genes related to an abnormality of the immune system (HP:0002715) and immune checkpoint genes (*2*); 4) had <1% allele frequency in the Genome Aggregation Database (gnomAD), Exome Variant Server, 1000 Genomes Project Consortium, dbSNPs; and 5) absent from the Thai reference exome (T-Rex) variant database (*3*). The candidate variants were called novel if they were not noted in the 1000 Genomes Project, gnomAD, and in-house databases.

PBMC Isolation

PBMCs from whole blood were isolated by density gradient centrifugation by using Lymphoprep (STEMCELL Technologies, https://www.stemcell.com) at 1,500 rpm for 30 min at

room temperature (no deceleration force). Isolated PBMCs were washed with RPMI 1640 Medium (GIBCO, Thermo Fisher–Life Technologies Corporation, https://www.thermofisher.com) supplemented with 10% fetal bovine serum (FBS) and cryopreserved in 10% dimethyl sulfoxide (DMSO) in FBS for further experiments.

T Cell Phenotyping

PBMCs were stained with monoclonal antibodies, including 7-AAD viability staining solution, anti-CD3-PE/Cy7, anti-CD4-AF700, anti-CD8-APC, anti-TIGIT- PE/Dazzle 594, and anti-CD69-BV650 (Biolegend, https://www.biolegend.com). After washing twice, cells were fixed and acquired by CytoFLEX flow cytometer (Beckman Coulter Life Sciences, https://www.beckmancoulter.com). All data were analyzed using Flowjo X software (https://www.flowjo.com).

T cell Functional Analysis

PBMCs were cultured in RPMI 1640 medium supplemented with 10% FBS and 1% penicillin-streptomycin (GIBCO). One million PBMCs were seeded in U-bottomed 96 well-plate (Thermo Fisher Scientific) and stimulated with or without anti-CD3/CD28-coupled beads (Thermo Fisher Scientific) for 24 hours. Brefeldin A (Biolegend) was added for the final 5 hours of incubation for intracellular cytokine detection. After the incubation period, cells were harvested and stained with antibodies for surface markers, including 7-AAD viability staining solution, anti-CD3-PE/Cy7, anti-CD4-AF700, anti-CD8-APC, anti-TIGIT-PE/Dazzle 594, and anti-CD69-BV650. Next, cells were fixed and permeabilized by BD Cytofix/Cytoperm (BD Biosciences, https://www.bdbiosciences.com), the intracellular staining was performed with fluorescently labeled antibodies including anti-TNFα-pacific blue, anti-IFNγ-PE, and anti-IL2-BV605 for 1 hour on ice. After washing twice, cells were fixed and acquired by CytoFLEX flow cytometer (Beckman Coulter Life Sciences, https://www.beckmancoulter.com), and all data were analyzed by using Flowjo X software. All antibodies were purchased from Biolegend.

Cytokine Measurement by Bio-Plex Multiplex Immunoassay System

Bio-Plex Pro Human Cytokine 27-plex Assay (Bio-Rad, https://www.bio-rad.com) was performed to investigate cytokine and chemokine levels, including FGF basic, Eotaxin, G-CSF, GM-CSF, IFN-γ, IL-1β, IL-1ra, IL-2, IL-4, IL-5, IL-6, IL-7, IL-8, IL-9, IL-10, IL-12 (p70), IL-13, IL-15, IL-17A, IP-10, MCP-1 (MCAF), MIP-1α, MIP-1β, PDGF-BB, RANTES, TNF-α, and

VEGF (Appendix Figure 4), in blood serum of healthy subjects and patients. Fifty microliters of diluted serum were used for the assay following the manufacturer's instructions. Levels of cytokines were measured by Bio-Plex 200 Systems (BioRad), and the data were analyzed using Bio-Plex Manager software (BioRad).

References

1. Ittiwut R, Sengpanich K, Lauhasurayotin S, Ittiwut C, Shotelersuk V, Sosothikul D, et al. Clinical and molecular characteristics of Thai patients with *ELANE*-related neutropaenia. J Clin Pathol. 2022;75:99–103. PubMed https://doi.org/10.1136/jclinpath-2020-207139

2. Hu FF, Liu CJ, Liu LL, Zhang Q, Guo AY. Expression profile of immune checkpoint genes and their roles in predicting immunotherapy response. Brief Bioinform. 2021;22:bbaa176. PubMed https://doi.org/10.1093/bib/bbaa176

3. Shotelersuk V, Wichadakul D, Ngamphiw C, Srichomthong C, Phokaew C, Wilantho A, et al. The Thai reference exome (T-REx) variant database. Clin Genet. 2021;100:703–12. PubMed https://doi.org/10.1111/cge.14060

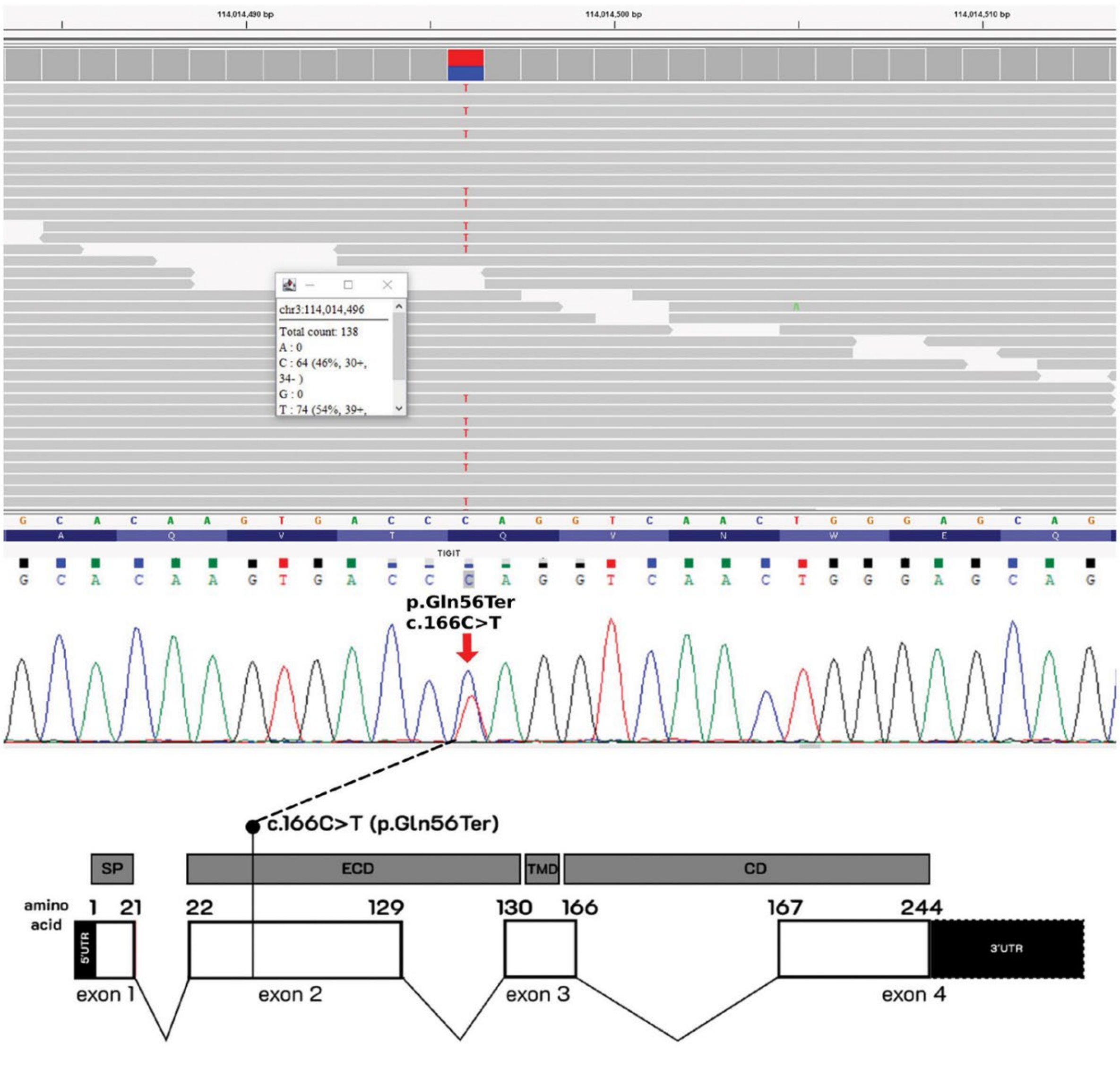

Appendix Figure 1. Whole-exome sequencing of *TIGIT* nonsense variant in a patient with severe COVID-19 infection, Thailand. Whole-exome sequencing of 138 read depths showed a nonsense *TIGIT*:c.166C>T (p.Gln56Ter) variant (64 reads with the reference C allele and 74 reads with alternative T allele) (Upper panel), compared with the direct sequencing electropherogram (middle panel). This variant was in exon 2 of the *TIGIT* gene, which causes truncation from the ECD to the entire polypeptide (bottom panel). ECD; extracellular domain; *TIGIT*, T cell immunoglobulin and ITIM domain gene.

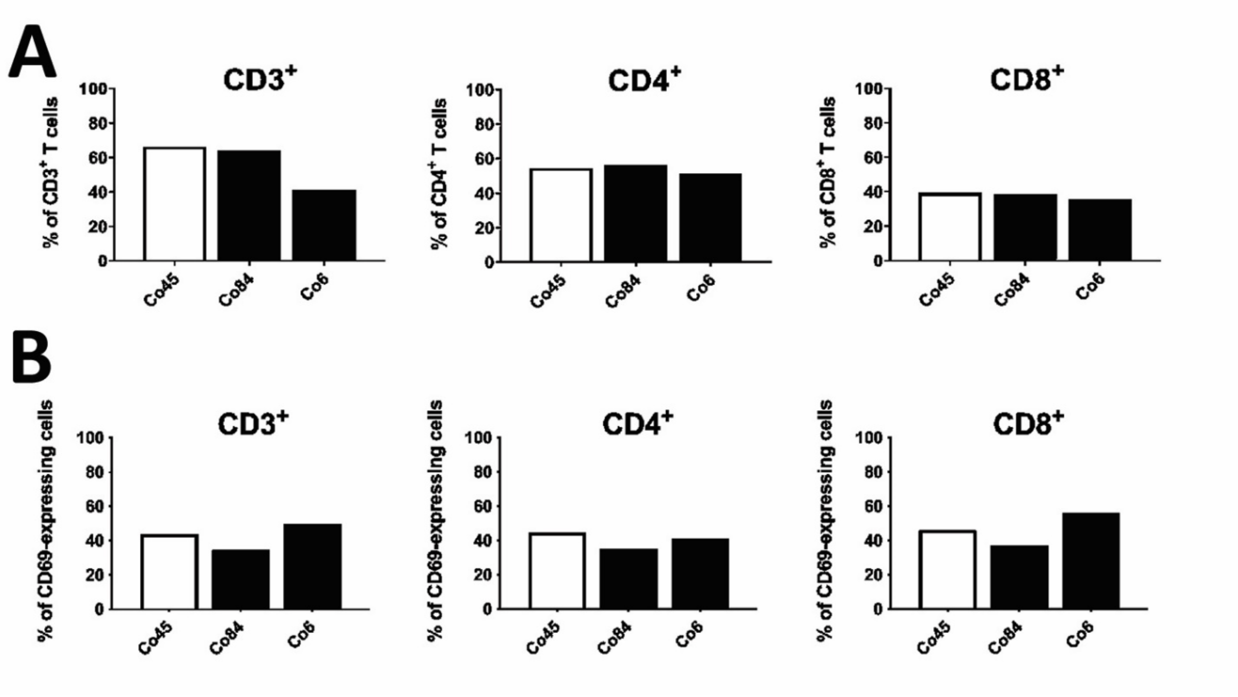

Appendix Figure 2. Measurement of T cell frequencies and activation of *TIGIT* nonsense variant in a patient with severe COVID-19 infection, Thailand. Co45 is the patient with *TIGIT* variation; Co84 and Co6 are the patients without the variation. A) We evaluated PBMCs for frequencies of $CD3^+$, $CD4^+$, and $CD8^+$ T cells. B) We stimulated PBMCs of the 3 patients with anti-CD3/CD28-coupled beads for 24 hours and measured the frequencies of $CD69^+CD3^+$, $CD69^+CD4^+$, and $CD69^+CD8^+$ T cells by flow cytometry. CD, cytoplasmic domain; PBMCs, peripheral blood mononuclear cells; *TIGIT*, T cell immunoglobulin and ITIM domain gene.

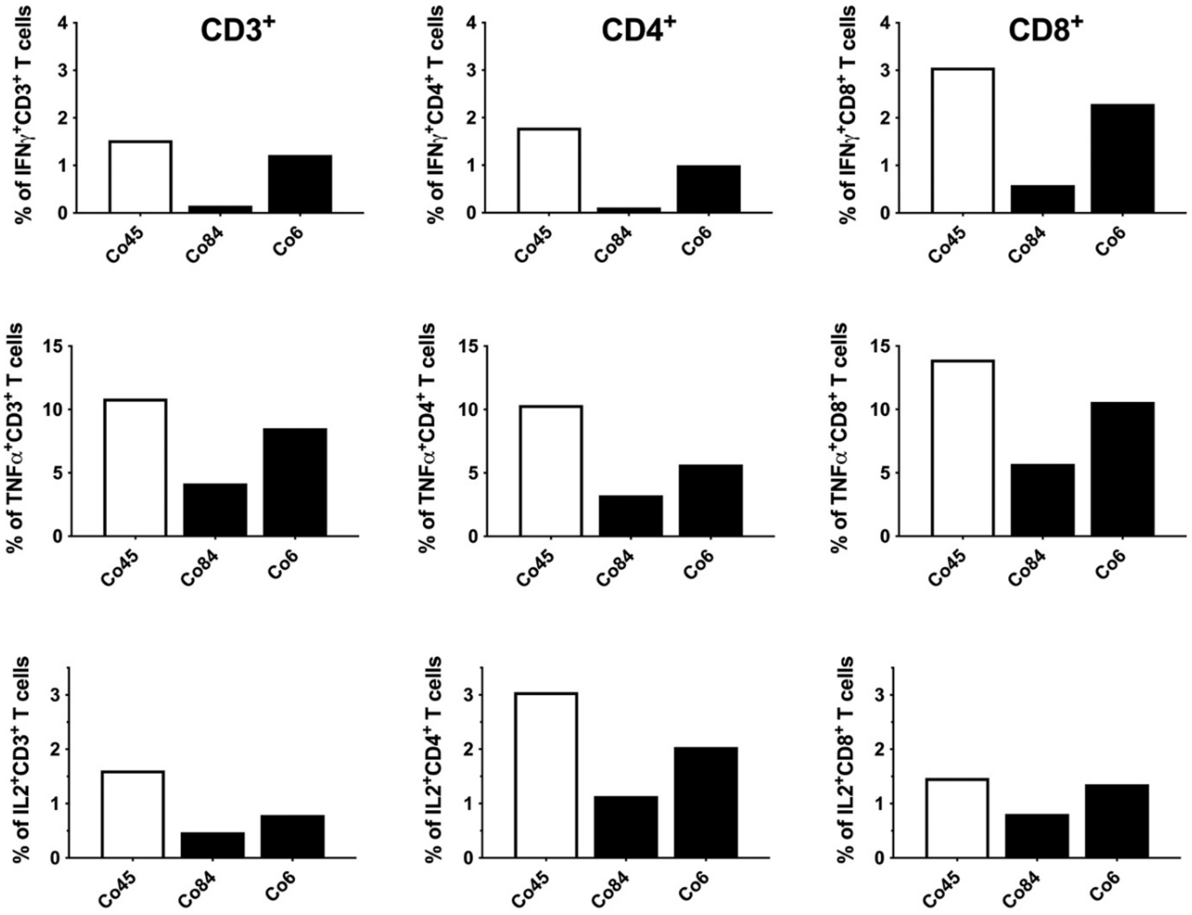

Appendix Figure 3. Measurement of T cell frequencies and activation of *TIGIT* nonsense variant in a patient with severe COVID-19 infection, Thailand. Co45 is the patient with *TIGIT* variation; Co84 and Co6 are the patients without the variation. We stimulated PBMCs with beads coupled with anti-CD3/CD28 for 24 hours and assessed by measuring IFN_{γ} (upper panel), TNF_{α} (middle panel), and IL-2 production (lower panel) in $CD3^+$, $CD4^+$, and $CD8^+$ T cells. CD, cytoplasmic domain; IFN_{γ}, interferon gamma; IL-2, lymphokine, interleukin 2; *TIGIT*, T cell immunoglobulin and ITIM domain gene; TNF_{α}, tumor necrosis factor alpha.

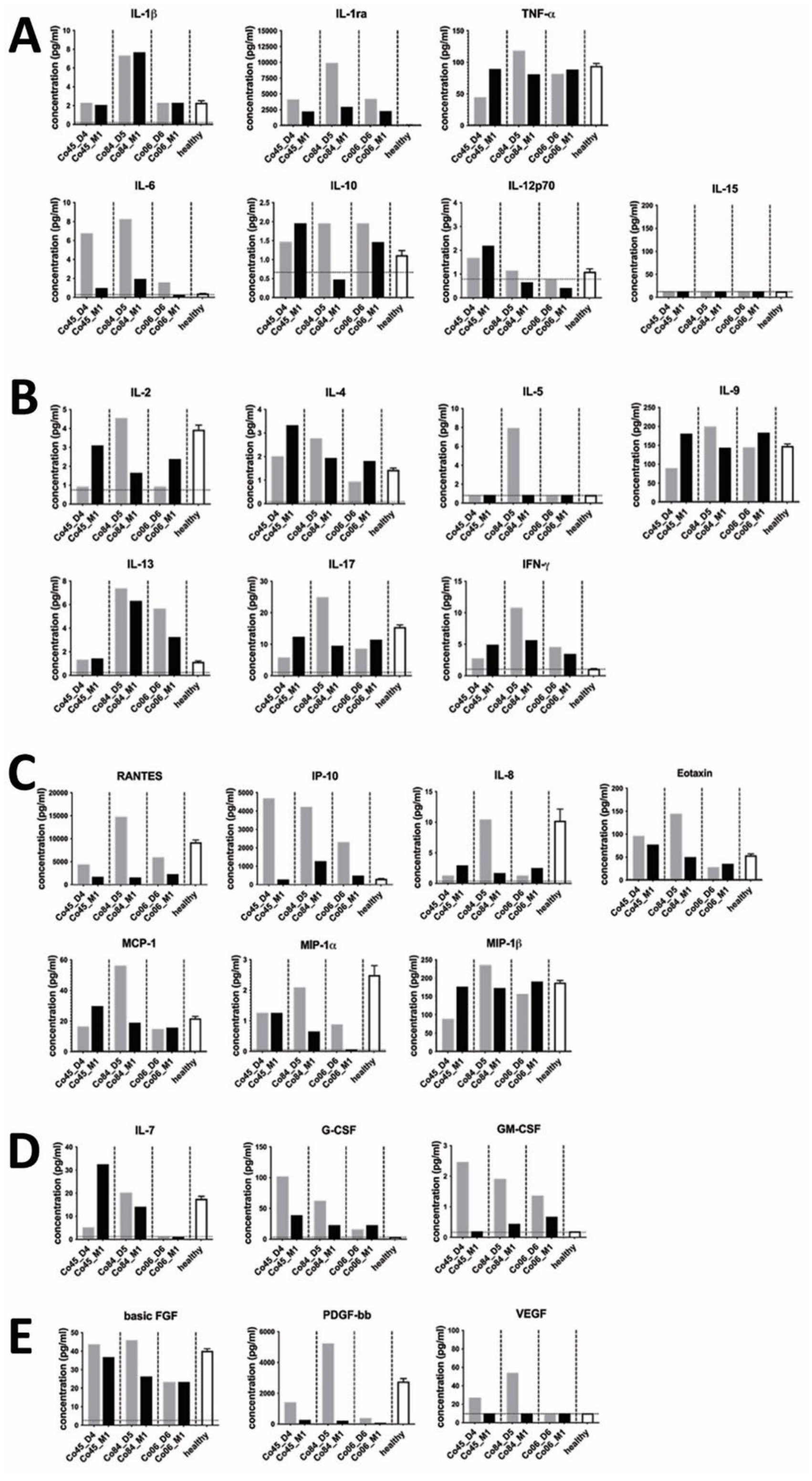

Appendix Figure 4. Measurement of cytokine and chemokine levels in a patient with *TIGIT* nonsense variant and severe COVID-19 infection, Thailand. We compared levels from the patient (Co45) with *TIGIT* variation against patients without the variation (Co84 and Co06) and against 40 healthy controls. Cytokine

and chemokine levels in serum of patients at acute COVID-19 infection phase (day 4, 5, or 6) and at 1 month after recovery were assessed comparing to healthy controls by using Bio-Plex Pro Human Cytokine 27-plex Assay (Lumninex, https://www.luminexcorp.com). Dotted lines indicate assay sensitivity, limit of detection (LOD). A) Cytokines in innate immunity; B) cytokines in adaptive immunity; C) chemokines; D) hematopoietic cytokines; E) growth factors. D4, day 4; D5, day 5; D6, day 6; FGF, fibroblast growth factor; G-CSF, granulocyte colony-stimulating factor; GM-CSF, granulocyte-macrophage colony-stimulating factor; IFN_{γ}, interferon gamma; IL, lymphokine, interleukin; M1, month 1; MCP, monocyte chemoattractant protein; MIP, macrophage inflammatory protein; PDGF, platelet-derived growth factor; *TIGIT*, T cell immunoglobulin and ITIM domain gene; VEGF, vascular endothelial growth factor.
